# Long-Term Disturbed Expression and DNA Methylation of SCAP/SREBP Signaling in the Mouse Lung From Assisted Reproductive Technologies

**DOI:** 10.3389/fgene.2021.566168

**Published:** 2021-06-24

**Authors:** Fang Le, Ning Wang, Qijing Wang, Xinyun Yang, Lejun Li, Liya Wang, Xiaozhen Liu, Minhao Hu, Fan Jin, Hangying Lou

**Affiliations:** ^1^Center of Reproductive Medicine, Zhejiang University School of Medicine Women’s Hospital, Hangzhou, China; ^2^Key Laboratory of Reproductive Genetics, Ministry of Education, Hangzhou, China

**Keywords:** assisted reproductive technologies, SCAP/SREBP, mice, DNA methylation, chronic lung diseases

## Abstract

Assisted reproductive technology (ART) has been linked to cholesterol metabolic and respiratory disorders later in life, but the mechanisms by which biosynthetic signaling remain unclear. Lung inflammatory diseases are tightly linked with the sterol regulatory element-binding protein (SREBP) and SREBP cleavage-activating protein (SCAP), but this has not been shown in an ART offspring. Here, mouse models from a young to old age were established including *in vitro* fertilization (IVF), intracytoplasmic injection (ICSI), and *in vivo* fertilized groups. In our results, significantly higher plasma levels of CRP, IgM, and IgG were identified in the aged ICSI mice. Additionally, pulmonary inflammation was found in four aged ART mice. At three weeks, ART mice showed significantly downregulated levels of *Scap*, *Srebp-1a*, *Srebp-1c*, and *Srebf2* mRNA in the lung. At the same time, significant differences in the DNA methylation rates of *Scap*-*Srebfs* and protein expression of nuclear forms of SREBPs (nSREBPs) were detected in the ART groups. Only abnormalities in the expression levels of *Srebp-1a* and *Srebp-1c* mRNA and nSREBP1 protein were found in the ART groups at 10 weeks. However, at 1.5 years old, aberrant expression levels and DNA methylation of SCAP, SREBP1, and SREBP2, and their associated target genes, were observed in the lung of the ART groups. Our results indicate that ART increases long-term alterations in SCAP/SREBP expression that may be associated with their aberrant methylation status in mouse.

## Introduction

*In vitro* fertilization (IVF) and other assisted reproductive technologies (ARTs) offer hope to subfertile couples worldwide. To date, more than 7 million babies have been delivered by ART worldwide, and every year, there is a sign that this trend is increasing (Novakovic et al., 2019). Although most ART babies and children are healthy, some studies are still concerned on the effects ART procedures may have on the long-term health of these children (Berntsen et al., 2019). Researches into the “developmental origins of health and disease” (DOHaD) in humans have demonstrated that exposures during early development (pre-conceptional, *in utero*, and early post-natal periods) can increase the risk of disease, particularly cardiovascular, metabolic, and respiratory disorders, later in life (Safi-Stibler and Gabory, 2020). Children born following ART may be at an increased risk of adult health problems, in part because of the laboratory techniques used, such as ovarian stimulation, embryo culture, embryo frozen, and intracytoplasmic sperm injection (ICSI) (Gluckman et al., 2008; Padhee et al., 2015; Feuer and Rinaudo, 2016). In addition to the possible risks posed by these techniques, studies have demonstrated adverse perinatal outcomes after ART (Dhalwani et al., 2016; Vrooman and Bartolomei, 2017; Maheshwari et al., 2018), which themselves can have consequences for adult heath. For instance, preterm birth, low birth weight, and being small for gestational age, which are well documented to be increased in children conceived by ART, have been associated with cardiometabolic disturbances in adulthood (Chen et al., 2012) and diminished lung function (Duijts, 2012).

Increased fasting glucose levels, blood lipid levels, adiposity, and blood pressure have been found in ART-conceived children (Valenzuela-Alcaraz et al., 2013; Lou et al., 2014; Meister et al., 2018). Moreover, in our recent and other long-term studies, ART induced the potential high risk of fatty liver in adulthood and resulted in an abnormal lipid metabolism in aged mice (Gu et al., 2018; Le et al., 2019). Emerging studies have indicated that dyslipidemia is often associated with lung disease (Yao et al., 2016). However, the long-term effects of ART on respiratory function remain poorly defined due to the relatively short time ART has been developed. Recent studies have suggested an association of IVF and ICSI with asthma in ART offsprings (Carson et al., 2013; Kuiper et al., 2015; Lewis et al., 2017). An increase in respiratory atopy was also reported in the IVF-conceived singletons compared with the controls (Halliday et al., 2014). Thus, there is a need to know whether adverse respiratory health outcomes later in life are associated with ART.

Persons with an impaired lung function have been found to have higher levels of cholesterol, suggesting a critical importance of alveolar cholesterol homeostasis in the normal lung physiology (Whitsett and Weaver, 2002). Cholesterol is essential for type II cell function; however, excessive amounts of cholesterol impair surfactant function. The transcriptional mechanisms regulating cholesterol synthesis have been shown to be dependent on the transcription factors sterol regulatory element-binding proteins (SREBPs) in the respiratory epithelium (Zhang et al., 2004; Bridges et al., 2014). Three distinct SREBP isoforms, SREBP-1a, -1c, and -2, are encoded by *Srebf1* and *Srebf2*. When intracellular cholesterol levels are abundant, SREBPs (SREBP1 and SREBP2) are held in the endoplasmic reticulum (ER) complexed to the sterol-sensing protein SREBP cleavage-activating protein (SCAP), which prevents the proteolytic generation of the transcriptionally active nuclear forms of SREBPs (nSREBPs), thereby limiting the transcription of SREBP target genes including the lipogenic and cholesterogenic genes, such as low density lipoprotein receptor (LDLR), acetoacetyl CoA synthetase (AACS), sterol 14α-demethylase (CYP51), farnesyl diphosphate synthase (FDPS), and 3-hydroxy-3-methyl-glutaryl-CoA reductase (HMGCR) (Ericsson et al., 1996; Horton et al., 2002; Harding et al., 2005). Deletion of *Scap* in the adult mouse lung inhibited SREBP activity in respiratory epithelial cells, resulting in an altered pulmonary lipid homeostasis (Besnard et al., 2009). Recent study reveals that, in addition to controlling cholesterol biosynthesis, SCAP-SREBP2 also serves as a signaling hub integrating cholesterol metabolism with inflammation in macrophages (Guo et al., 2018). Aberrant SREBP activity in respiratory epithelial cells was linked with lipotoxicity-related lung inflammation and tissue remodeling in adult mice (Plantier et al., 2012). Furthermore, an aberrant epigenetic profile may be the potential mechanism of adult disease risk in ART-conceived offspring (El et al., 2017; Safi-Stibler and Gabory, 2020). Data demonstrated that DNA methylation may be a part of the biological processes underlying lung function (Lepeule et al., 2012). Hence, we hypothesized that the periconception and early intrauterine exposures associated with ART lead to poorer physical health outcomes, perhaps through epigenetic changes in SCAP/SREBP, resulting in a higher prevalence of chronic lung illness.

Due to the improvement in respiratory function at a young age and dyslipidemia in ART-conceived individuals, there is a need to establish whether ART is associated with lung inflammation later in life. In our recent study, abnormalities in the blood lipids were identified in the ART-conceived elderly mice (Le et al., 2019). Thus, this present study was designed to ascertain whether conception by ART is associated with respiratory diseases later in life through the establishment of mouse models of conception including IVF, ICSI, and *in vivo*. Furthermore, given the mounting evidence for a role of the SCAP/SREBP pathway and epigenetic regulation in chronic lung disorders, we defined the dynamic roles of the SCAP/SREBP pathway and the methylation status of its components in the lungs of ART-conceived mice from a young to old age.

## Materials and Methods

### Animals

C57BL/6J female (6–8 weeks) and male mice (10–12 weeks) were used as oocyte and sperm donors in this study. This study was approved by the Ethics Committee of Zhejiang University Animal Care. All of the animals were housed with a 12 h light/dark cycle at 25 ± 0.5°C and 50–60% humidity. The C57BL/6J female mice were randomly divided into the ICSI, IVF, and *in vivo* groups. All of the experiments were conducted between 09:00 and 17:00 to minimize the circadian influences.

### Production of IVF-, ICSI-, and *in vivo*-Conceived Mice

#### IVF and ICSI Groups

First, C57BL/6J female mice were superovulated with an intraperitoneal injection of 7.5 IU of pregnant mare serum gonadotropin (PMSG), and 48 h later, with an intraperitoneal injection of 7.5 IU of human chorionic gonadotropin (hCG), as previously reported (Le et al., 2019). Then, the cumulus-oocyte complexes were collected after 15 h post-hCG administration in human tubal fluid (HTF) medium, which contained 10% synthetic serum substitute (SSS) (Irvin Scientific, United States) at 37°C. Lastly, cumulus-oocyte complexes were then either transferred into a fertilization drop (for the IVF group) or into a dispersion drop (for the ICSI group). Before using for insemination or microinjection, sperm were capacitated for 1.5 h at 5% CO_2_ in air at 37°C which were obtained from the epididymis of C57BL/6J males.

As previously described (Le et al., 2019), in the IVF group, oocyte-cumulus cell complexes were added into a drop in which the final sperm concentration was approximately 1–2.5 × 10^6^ ml^–1^. The embryos were cultured in fresh 10% SSS HTF at 5% CO_2_ in air at 37°C after fertilization. Then, 24 h later, 2-cell-stage embryos were obtained from the IVF group. In the ICSI group, metaphase II (M II) oocytes were fertilized by ICSI. Sperm heads were singly injected into each oocyte as previously described (Le et al., 2019). Injected oocytes were cultured in 10% SSS fresh HTF in humidified 5% CO_2_ at 37°C. Then, 24 h later, 2-cell-stage embryos were obtained from the ICSI group.

#### *In vivo* Group

In the *in vivo* group, C57BL/6J female mice were caged with male mice at a ratio of 1:1. The next day, we separated the female mice with vaginal plugs from the males. After 44 h, the 2-cell embryos for the *in vivo* group were obtained from the oviducts.

#### Embryo Transfer and Tissue Collection

ICR (Institute of Cancer Research) females (8–10 weeks old) were used as pseudopregnant recipients. After the females mated with the ICR vasectomized males (2:1), vaginal plugs were detected in the females which was considered day 0.5 of the pseudopregnancy. As previously shown (Le et al., 2019), 12–15 of 2-cell-stage embryos were transferred into the oviducts of a 0.5-d pseudo-pregnant foster mother in each group. The litters were redistributed on the day after birth to have litter sizes of six pups to ensure standardized nutrition and maternal care. In total, 36 *in vivo*-conceived, 32 IVF-conceived, and 32 ICSI-conceived mice were examined. The birth outcomes were shown in our recent published work (Le et al., 2019).

### Serum Analysis

At 1.5 years old, after the mice were euthanized, venous blood of aged mice was withdrawn. Plasma levels of C-reactive protein (CRP), immunoglobulin (Ig) G, and IgM were determined using commercially available specific ELISA kits according to the instructions provided by the manufacturers (Cusabio Company, Wuhan, China).

### Histological Analysis of Mouse Models

At 3 weeks (*n* = 10/group), 10 weeks (*n* = 10/group), and 1.5 years old (IVF, *n* = 12; ICSI, *n* = 12; *in vivo*, *n* = 16), lungs were excised and weighed. In addition, histopathological analyses were performed. Afterfixed in 4% paraform for 24 h, lungs were transfered to 70% ethanol. Individual lobes of biopsy material were placed in processing cassettes, dehydrated through a serial alcohol gradient, and embedded in paraffin wax. Lung tissue sections (5 μm) were stained with hematoxylin and eosin (HE). The slides were observed under a microscope, and five regions within the HE-stained sections were examined and scored at 200 × magnification. Morphological analysis of random fields from each section was performed by two experienced pathologists. Analysis of lung injury was scored on the basis of four parameters as previously described (Smith et al., 1997): (a) alveolar hemorrhage, (b) alveolar edema, (c) infiltration or aggregation of neutrophils in the airspace or the vessel wall, and (d) thickness of the alveolar wall.

### Real-Time Quantitative PCR (RT-qPCR)

Total RNA was extracted from the lung (*n* = 10/group) by using the RNAiso Plus (TaKaRa, Tokyo, Japan). Then, RT-qPCR was conducted as previously described (Le et al., 2019). The quantification of gene transcripts was conducted by RT-qPCR by using the SYBR^®^ Premix Ex Taq^TM^ (TaKaRa, Tokyo, Japan) in an ABI 7900 thermocycler. The primers of *Scap*, *Srebf2*, and their associated genes are shown in Supplementary Table 1. The primers of *Srebp-1a* and *Srebp-1c* were used as shown in the previous study (Besnard et al., 2009). The housekeeping gene *Gapdh* was used as the reference gene and was stably expressed between our treatment groups. The fold-change was calculated by using the comparative Ct method.

### Western Blotting Analysis

Western blot analysis of SCAP/SREBP was performed as previously described (Le et al., 2019). Briefly, aliquots (45 μg) of the nuclear and membrane fractions from three mice per group were subjected to SDS-PAGE on 8 or 10% gel. The separated samples were transferred to nitrocellulose membranes exposed to the rabbit anti-GAPDH antibody (1:1,000 dilution; Abcam, Inc.), mouse anti-SREBP1 antibody (1:1,000 dilution; Abcam, Inc.), and rabbit anti-SCAP/SREBP2 antibodies (1:1,000 dilution; Abcam, Inc.) for 2 h after electrophoresis. Then, the membranes were incubated in the DyLight^TM^ 680-Labeled goat anti-rabbit or anti-mouse IgG (H–L) (1:5,000 dilution; KPL, Gaithersburg, United States) for 1 h at room temperature. Lastly, the images were visualized by an Odyssey^®^ Imager (LI-COR, United States).

### Methylation-Specific PCR (MSP) and Pyrosequencing Analysis

As described previously, DNA (1 μg) was processed from the lung (*n* = 10/group) for bisulfite sequencing analysis using the EpiTect Bisulfite kit (Qiagen, Valencia, CA, United States). Primers for *Scap*, *Srebf1*, and *Srebf2* are shown in Supplementary Table 2. By using the PSQ 96 ID system, the methylation-specific PCR and pyrosequencing were conducted (Le et al., 2019). The mean ± *SD* was calculated for DNA methylation rates.

### Statistical Analyses

Data were analyzed using a software (SPSS, Version 16. Chicago, IL, United States). The mean ± *SD* was shown for data. Between-group comparisons were made by one-way analysis of variance (ANOVA). When required, non-parametric tests were used as indicated. The significance level was set at *P* < 0.05.

## Results

### Organ Weight, Histological, and Plasma Analysis of the ART Mice

As shown in Table 1, there was no difference in the body weight and lung weight between the ART groups and the *in vivo* group at young (3 weeks), adult (10 weeks), and old (1.5 years) ages. However, as shown in Table 2, the aged ICSI mice showed significantly higher levels of CRP, IgG, and IgM than the *in vivo* mice (*P* < 0.05). Moreover, in our recent paper (Le et al., 2019), higher levels of cholesterol and low density lipoprotein-cholesterol (LDL-C) and lower levels of high-density lipoprotein-cholesterol (HDL-C) and apolipoprotein-A1 (Apo-A1) were found in the aged ICSI mice.

**TABLE 1:** Comparison body and organ weight of ART and *in vivo* mice at three growth stages.

	*In vivo*	IVF	ICSI	*P*
Numbers	36	32	32	>0.05
Sex ratio	1:1	1:1	1:1	>0.05
**Body weight (g)**				
3 weeks	11.33 ± 0.78	10.68 ± 1.09	11.15 ± 0.52	>0.05
10 weeks	26.16 ± 3.78	26.06 ± 2.85	26.20 ± 4.06	>0.05
1.5 years	26.03 ± 3.82	25.04 ± 3.56	25.27 ± 2.28	>0.05
**Lung weight (g)**				
3 weeks	0.11 ± 0.01	0.11 ± 0.02	0.12 ± 0.03	>0.05
10 weeks	0.15 ± 0.01	0.14 ± 0.01	0.14 ± 0.01	>0.05
1.5 years	0.21 ± 0.07	0.26 ± 0.15	0.23 ± 0.10	>0.05

**TABLE 2:** Plasma analysis of the aged mice conceived by ART and *in vivo.*

	*In vivo*	IVF	ICSI	*P*
Numbers	16	12	12	>0.05
Sex ratio	1:1	1:1	1:1	>0.05
*N* (inflammation)	N/A	2	2	>0.05
CRP (mg/L)	3.81 ± 0.53	4.34 ± 0.68	4.59 ± 0.54	<0.01
IgG (mg/mL)	6.92 ± 0.12	7.09 ± 0.37	7.36 ± 0.53	<0.05
IgM (mg/mL)	1.05 ± 0.04	1.19 ± 0.14	1.25 ± 0.13	<0.01

*N, Numbers of lung inflammation.*

*P < 0.05: ICSI vs. in vivo (ANOVA), P < 0.01: ICSI vs. in vivo (ANOVA).*

**FIGURE 1:**
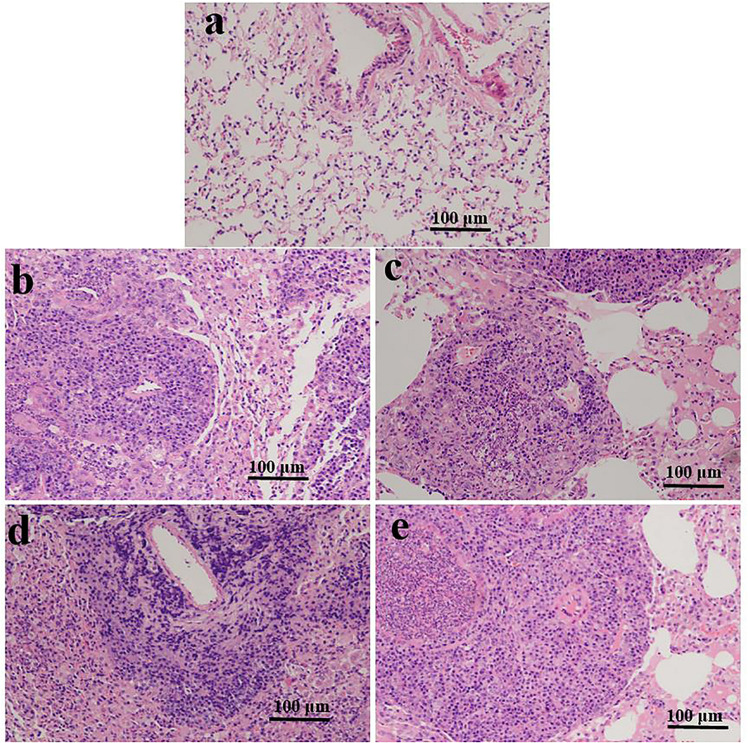
Representative photomicrographs of HE-stained lung sections are shown for ART- and *in vivo*-conceived aged mice. The lungs of an *in vivo*-conceived female **(a)**, an IVF-conceived female **(b)**, an IVF-conceived male **(c)**, and an ICSI-conceived male **(d,e)**. Histology analysis are based on alveolar edema, hemorrhage, infiltration, or aggregation of neutrophils in the airspace or the vessel wall and thickness of the alveolar wall. Original magnification × 200. In **(b–e)**, lung architecture was compromised and a large number of inflammatory cells infiltrated in the alveolar interstitium.

Histology analysis based on alveolar edema, hemorrhage, infiltration, or aggregation of neutrophils in the airspace or the vessel wall and thickness of the alveolar wall. Representative images of lung sections were analyzed (HE staining; Figure 1). At 3 and 10 weeks, we found no histological changes in the lungs of the ART-conceived mice compared to those in the *in vivo* mice. Although no statistical differences were found between the ART and *in vivo* groups, as shown in Figures 1b–e, lung architecture was compromised in two ICSI males and two IVF mice (one female and one male). In addition, infiltration of a large number of inflammatory cells was found in the alveolar interstitium of the ART group (Figures 1b–e) as compared with the *in vivo* group (Figure 1a).

### Long-Term Changes in the mRNA Expression Levels of *Scap*/*Srebp* in the Lung

We assessed the effects of ART on the mRNA expression of *Scap*/*Srebp* in the lung from a young to an old age.

**FIGURE 2:**
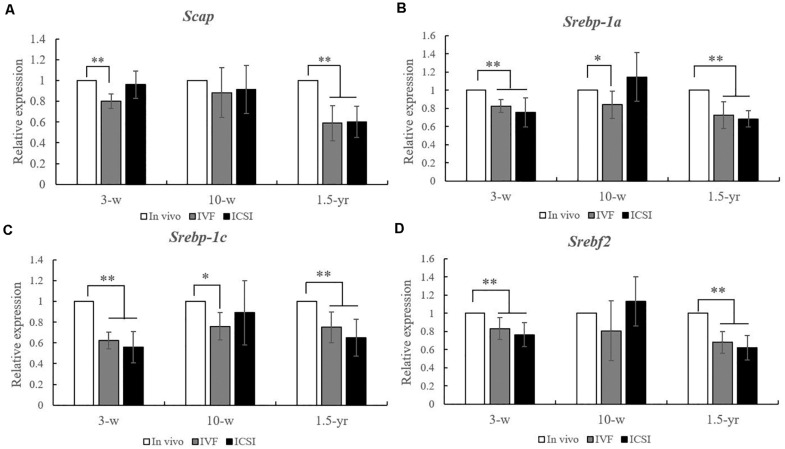
Expression level analysis of *Scap*, *Srebp-1a*, *Srebp-1c*, and *Srebf2* mRNA in the lungs from the ICSI, IVF, and *in vivo* groups (*n* = 10/group). mRNA expression level analysis at 3, 10 weeks, and 1.5 years of age by real-time quantitative PCR (RT-qPCR). **(A)** mRNA expression level of S*cap*. **(B)** mRNA expression level of *Srebp-1a*. **(C)** mRNA expression level of *Srebp-1c*. **(D)** mRNA expression level of of *Srebf2.* The relative expression levels represent the amount of expression normalized to *Gapdh* expression. Data concerning the relative amount was calculated by the 2^–ΔΔ*Ct*^ method. Mean ± *SD* values are plotted. Between-group comparisons were made by one-way analysis of variance (ANOVA). *P < 0.05, **P < 0.01. 3-w, 3 weeks; 10-w, 10 weeks; 1.5-yr, 1.5 years old.

As shown in Figures 2A–D, at 3 weeks, significantly lower expression levels of *Scap*, *Srebp-1a*, *Srebp-1c*, and *Srebf2* mRNAs were found in the lung of IVF- and ICSI-conceived mice compared with that in the *in vivo* mice (0.5–0.8-fold, *P* < 0.01). At 10 weeks, compared with the expression levels in the *in vivo* mice, only the expression levels of *Srebp-1a* and *Srebp-1c* mRNAs were statistically lower in the IVF mice (0.7- and 0.8-fold, respectively, *P* < 0.05). However, at 1.5 years old, statistically lower expression levels of *Scap*, *Srebp-1a*, *Srebp-1c*, and *Srebf2* mRNA were found in the IVF- and ICSI-conceived aged mice compared with those in the *in vivo* group (0.5–0.7-fold, *P* < 0.01).

### Long-Term Changes in the DNA Methylation Status of *Scap*/*Srebf* in the Lung

**FIGURE 3:**
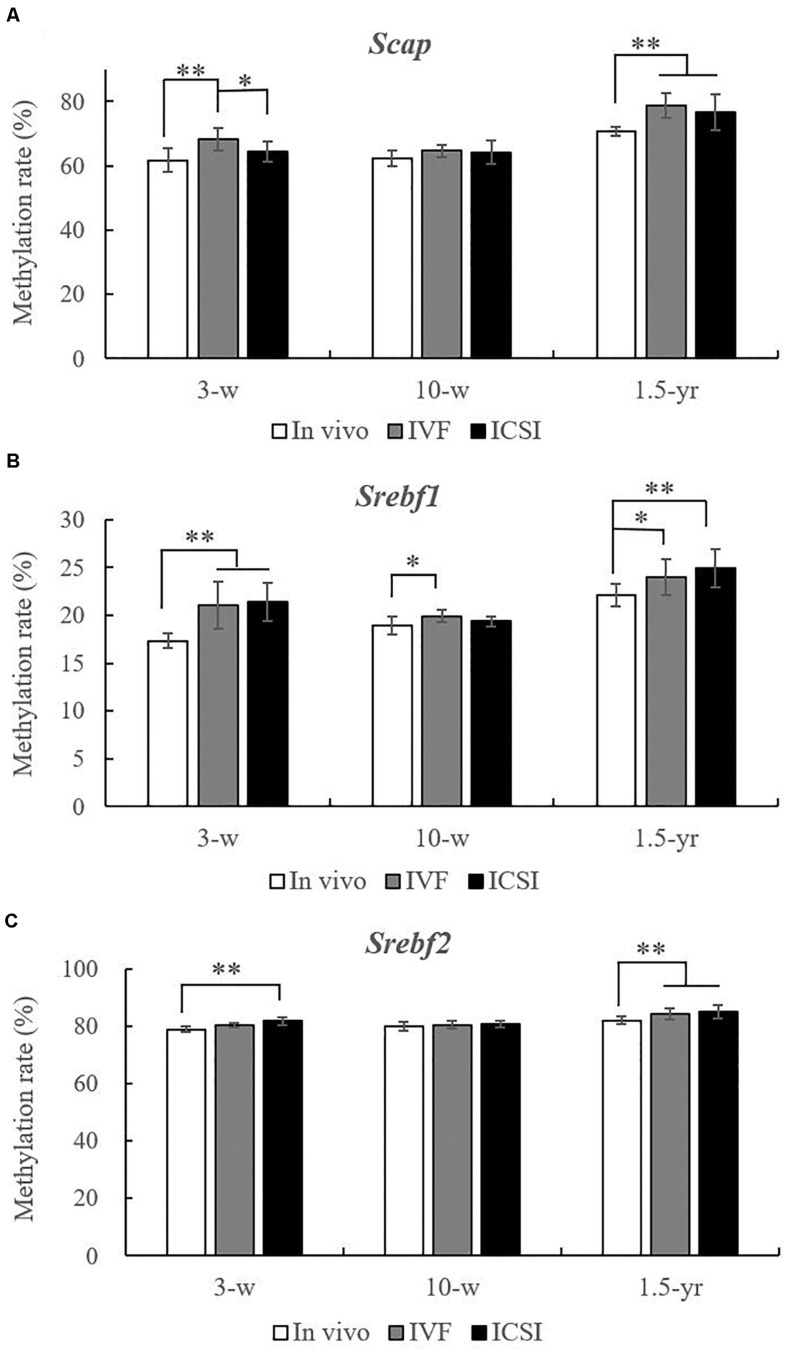
Statistical methylation analysis of S*cap*, *Srebf1*, and *Srebf2* in the IVF, ICSI, and *in vivo* groups (*n* = 10/group). **(A)** DNA methylation rate of S*cap* at 3 weeks, 10 weeks, and 1.5 years of age. **(B)** DNA methylation rate of *Srebf1* at 3 weeks, 10 weeks, and 1.5 years of age. **(C)** DNA methylation rate of *Srebf2* at 3 weeks, 10 weeks, and 1.5 years of age. Mean ± SD values are plotted. Between-group comparisons were made by one-way analysis of variance (ANOVA).*P < 0.05, **P < 0.01. 3-w, 3 weeks; 10-w, 10 weeks; 1.5-yr, 1.5 years old.

As shown in Figures 3A–C, at 3 weeks, the methylation rate of *Scap* was significantly higher in the IVF group (68.37 ± 3.49%) than in the *in vivo* group (61.80 ± 3.72%) and ICSI group (64.45 ± 2.99%). Moreover, compared with the *in vivo* group (17.36 ± 0.75%), the *Srebf1* showed a significantly higher methylation rate in the IVF (21.04 ± 2.46%) and ICSI (21.42 ± 2.04%) groups (*P* < 0.01). A higher methylation rate of *Srebf2* was also found in the ICSI group than in the *in vivo* group (81.76 ± 1.27% vs. 79.00 ± 0.83%, *P* < 0.01). At 10 weeks, only the methylation rate of *Srebf1* was significantly higher in the IVF group compared with that in the *in vivo* group (19.91 ± 0.64% vs. 18.91 ± 0.95%, *P* < 0.05). There were no significant differences in the methylation rate of *Scap* and *Srebf2* among the three groups. But at 1.5 years old, the methylation rate of *Scap* in the ICSI group (76.65 ± 5.68%) and IVF group (78.77 ± 3.76%) was significantly higher than that in the *in vivo* group (70.67 ± 1.40%) (*P* < 0.05). In addition, ICSI and IVF mice had a significantly higher methylation rate than the *in vivo* group at the *Srebf1* and *Srebf2* CpG sites (*P* < 0.05).

### Long-Term Changes in the Protein Expression Levels of SCAP-SREBP in the Lung

**FIGURE 4:**
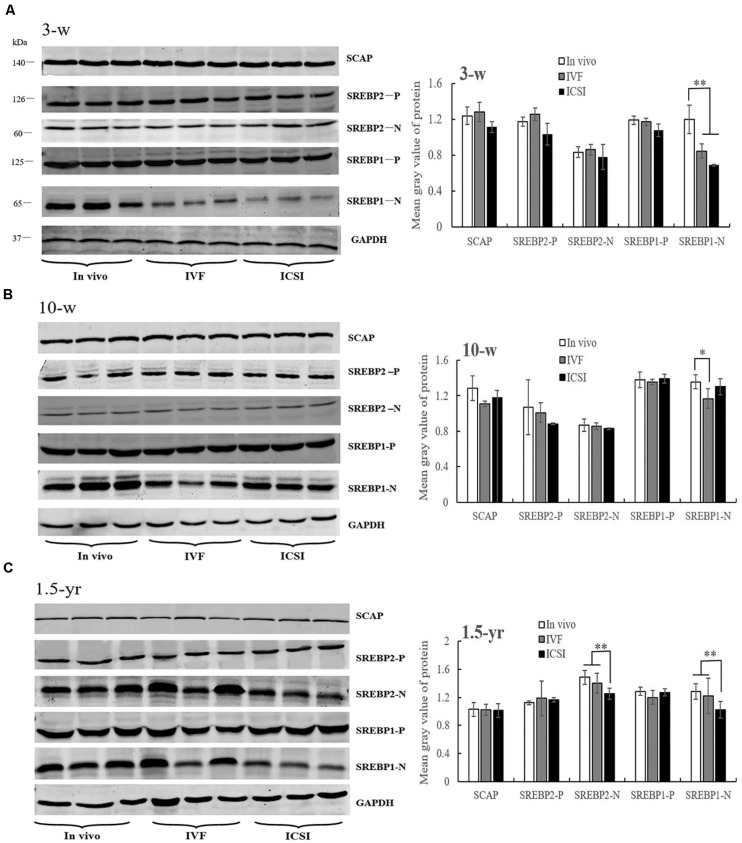
Western blotting analysis of membrane (SCAP and SREBP1/2-P) and nuclear extract (SREBP1/2-N) fractions in the ICSI, IVF, and *in vivo* mice. Bands of SCAP, SREBPs, and GAPDH and the mean gray value at 3 weeks **(A)**, 10 weeks **(B)**, and 1.5 years of age **(C)**. N, nuclear extract of SREBP; P, precursor form of SREBP. Mean ± *SD* values are plotted. Between-group comparisons were made by one-way analysis of variance (ANOVA). *P < 0.05, **P < 0.01. 3-w, 3 weeks; 10-w, 10 weeks; 1.5-yr, 1.5 years old.

At 3 weeks, IVF and ICSI mice showed a significant downregulation in the expression of nSREBP1 amounts compared with the *in vivo* group in the lung (Figure 4A, *P* < 0.01). There were no significant differences in other proteins, such as SCAP, the precursor of SREBP1 (pSREBP1) and SREBP2, among the groups (Figure 4A, *P* > 0.05). As shown in the Figure 4B, at 10 weeks, a significantly lower expression level of nSREBP1 amounts was found in the IVF mice compared to that in the *in vivo* mice (*P* < 0.05). There were no significant differences in the expression of other proteins among the three groups, such as SREBP2 and SCAP. At 1.5 years of age, significantly lower expressions of nSREBP1 and nSREBP2 amounts were found in the ICSI group when compared with the expression in other groups (*P* < 0.05). No expression differences were identified for SCAP and pSREBPs among the three groups (Figure 4C, *P* > 0.05).

### The mRNA Expression Changes of the nSREBP Associated Genes in the Lung of Aged ART-Conceived Mice

From the above protein results, significantly lower expression levels of nSREBPs were found in the lung of ICSI-aged mice. Thus, we further assessed the mRNA expression of nSREBP target genes in the lung at 1.5 years old. As shown in Supplementary Figure 1A, statistically lower expression levels of *Ldlr*, *Cyp51*, and *Fdps* mRNAs were found in the ICSI-conceived aged mice compared with those in the *in vivo* and IVF groups (0.5–0.8-fold, *P* < 0.01). There were no significant differences in the expression of *Aacs* and *Hmgcr* in the ICSI group. In addition, only the expression levels of *Aacs* mRNA were found in the IVF group as compared with the other groups (0.6-fold, *P* < 0.01).

Furthermore, lower amounts of nSREBPs not pSREBPs protein was found in the ICSI mice in our work. Studies show that nSREBP is released from the membrane by two sequential proteolytic cleavages by Site-1 protease (S1P, MBTPS1) and Site-2 protease (S2P, MBTPS2) (Yang et al., 2002). Thus, the mRNA expression levels of *Mbtps1* and *Mbtps2* were analyzed in the lung at old age. As shown in Supplementary Figure 1B, the expression levels of *Mbtps1* and *Mbtps2* mRNAs were significantly lower in the ICSI-conceived aged mice than in the IVF and *in vivo* conceived mice (0.3- and 0.7-fold, *P* < 0.01).

## Discussion

Given the increasing risk of respiratory disorders at a young age and dyslipidemia in an ART-conceived offspring (Lou et al., 2014; Kuiper et al., 2015; Lewis et al., 2017), there is a need to know whether these situations are associated with lung dysfunctions later in life. However, the long-term effects and the molecular mechanisms associated with respiratory disorders in ART-conceived individuals remain poorly defined. Thus, we sought to identify the role of the SCAP/SREBP pathway, a known signaling cascade associated with cellular lipid homeostasis, in the lung of ART-conceived mice from a young to an old age. In our study, pulmonary inflammation was found in four aged ART mice. In addition, significantly higher plasma levels of CRP, IgM, and IgG were identified in the aged ICSI mice. Our results detected that the SCAP/SREBP signaling expression was influenced by ART from a young age and was changed the most in the elderly. Moreover, we found that IVF and ICSI produced persistent changes in the SCAP/SREBP expression through the epigenetic regulatory mechanism of DNA methylation.

Impaired glucose tolerance, increased body fat and stiffness, and altered fatty acid composition have been reported in an IVF-conceived offspring (Ceelen et al., 2008; Scherrer et al., 2012; Feuer and Rinaudo, 2016; Vrooman and Bartolomei, 2017). Our recently and previously published papers also found that ART-conceived offspring, including IVF- or ICSI-conceived mice and human fetuses, had higher blood lipid levels across development, such as cholesterol, triglycerides, LDL, and apolipoproteins (Lou et al., 2014; Le et al., 2019). Previous data indicated that lipid storage diseases are often accompanied by chronic inflammation and fibrosing alveolitis (Minai et al., 2000). In our work, no histological changes in the lung were found in young or adult ART-conceived mice, but four aged ART mice showed a higher infiltration of inflammatory cells in the lung. Moreover, persons with an impaired lung function have been found to have higher levels of inflammatory markers, such as CRP and IL6. Inflammatory markers also may be correlated with inflammatory diseases in other organs, such as the liver, kidney, heart, pancreas, brain, and reproductive system. No inflammatory diseases were found in the liver, heart, and brain in our previously published work (Le et al., 2019). In the elderly, ICSI-conceived mice exhibited higher plasma levels of CRP, IgM, and IgG, which suggest that *in vitro* manipulations may be linked with an increased risk of chronic lung inflammation later in life. Additionally, a few recent studies of children indicated that those born after ART were more likely to be prescribed with anti-asthmatic medication, but the underlying duration of subfertility rather than an effect of treatment appeared to be the putative risk factor (Finnstrom et al., 2011; Kallen et al., 2013). In our mouse models, after excluding subfertility factors, we still observed higher inflammatory biomarker levels in the elderly ICSI mice, which may suggest that *in vitro* manipulations or ovarian stimulation could be the potential risk factors for the later occurrence of lung inflammation. Moreover, anatomopathological analysis of ICSI-conceived animals later in life showed an increase in the presence of solid tumors in the lungs compared to the analysis of the control animals (Fernandez-Gonzalez et al., 2008). Thus, as technology continues to improve, researchers will continue to uncover the effects of ART on later occurences of lung diseases.

The SCAP/SREBP pathway has previously been extensively studied in relation to cholesterol metabolism, lipogenesis, and glucose homeostasis (Yang et al., 2002). Data from both bioinformatics and mouse models have demonstrated important roles for SCAP/SREBP signaling in lung lipid homeostasis (Besnard et al., 2009; Plantier et al., 2012). For the first time, abnormal expression levels of *Srebp-1a*, *Srebp-1c*, *Srebf2*, and *Scap* mRNA were found in the lungs of ART-conceived mice both at a young and an elderly age, which suggests that the periconceptual manipulations of ART have a long-term influence on the gene expressions of SCAP/SREBP signaling. However, ART-conceived mice in our study only showed a decreased level of nSREBP1 and nSREBP1 amounts in the lung, which may be due to the complicated post-transcriptional, translation, and post-translational modifications of SCAP/SREBP (Greenbaum et al., 2003). Previous evidence indicated that miRNAs are involved in the regulation of SCAP/SREBP post-transcriptionally (Bridges et al., 2014). Furthermore, the SCAP/SREBP is also regulated by various post-translational modifications, such as acetylation, phosphorylation, and sumoylation (Cheng et al., 2018). Recent reports demonstrated that SREBP1 activation was shown to induce lipotoxicity that consequently extended SREBP-related pathology to include inflammation and fibrosis in the lung (Zhou et al., 2015; Shichino et al., 2019). In our results, lower mRNA transcription and protein levels of nSREBP1 were found from young, to adult, and to elderly life, which further indicate that SREBP1 may be particularly vulnerable to differential expressions because of early life manipulation. SREBP-1a and SREBP-1c with a different exon 1 are known as two isoforms of SREBP1. SREBP-1c is involved in fatty acid synthesis and lipogenesis, whereas SREBP1a are mainly involved in cholesterol synthesis (Besnard et al., 2009). Recent publications indicate that SREBP-1a also plays a crucial role in the inflammatory response in macrophages (Lee et al., 2018). Activation of SCAP/SREBP enhances lipogenesis causing pulmonary lipotoxicity in both alveolar type II epithelial cells and alveolar macrophages (Besnard et al., 2009; Guo et al., 2018). In our work, not only *Srebp-1c* but also *Srebp-1a* expressions were lower in the lung of ART-conceived mice, suggesting that both alveolar macrophages and epithelial cells may be responsible for their reduction. However, additional *in vitro* studies about this topic should be conducted in the future.

The SREBPs are synthesized as precursors (pSREBP) located in the ER membrane where it forms a complex with SCAP and insulin induced gene (INSIG). Two proteases cleave pSREBP in the Golgi to release the transcriptionally active nSREBP (Yang et al., 2002). The nSREBPs then translocate into the nucleus where it binds to sterol regulatory element (SRE) binding sites in the regulatory region of target genes (Goldstein et al., 2006). Lower amounts of nSREBPs not pSREBPs protein were found in our ICSI-conceived mouse models. The reason for the result may from the lower expression of *Scap*, *Mbtps1*, and *Mbtps2*, which processed pSREBP in the Golgi leading to decreased levels of active nSREBPs. In our results, lower expression levels of the nSREBP target genes, such as *Ldlr*, *Cyp51*, *Aacs*, and *Fdps*, were found in the lung of ART-aged mice, especially in the ICSI group, which may be associated with an alveolar cholesterol imbalance in lung physiology and result in lung diseases. Furthermore, nSREBP is regulated by other post-translational mechanisms (Cheng et al., 2018). Together, these results indicate that ART induced long-term nSREBPs perturbation of their activity, which may cause a potentially higher risk of lung inflammation later in life.

Given the evidence for a strong influence of *in vitro* exposures on epigenetic regulation, epigenetic changes may be one of the factors that explain the increasing prevalence of asthma, chronic obstructive pulmonary disease, and interstitial lung disease (Lepeule et al., 2012; Safi-Stibler and Gabory, 2020). Experimental studies are beginning to point to the role of the SCAP/SREBP pathway in the development of lung diseases in animals (Bridges et al., 2014). Our group hypothesized that aberrant DNA methylation of the SCAP/SREBP pathway at different growth stages may link with the potentially higher risk of later lung inflammation in ART-conceived mice. Hence, we investigated the DNA methylation of SCAP/SREBP in the lung from a young to old age. At young, ART mice showed higher methylation rates of *Scap*, *Srebf1*, and *Srebf2*, which is in accordance with a decrease in the expression of each of these transcripts. However, in adult ART-conceived mice, only *Srebf1* showed a significantly higher methylation rate than those in the *in vivo* mice, which suggests that manipulation during embryo development may alter the methylation status of specific genes. With an advanced age, the ART mice showed higher methylation rate of all the genes, including *Scap*, *Srebf1*, and *Srebf2*, which indicates a specific epigenetic variability that is associated with aging. Consistent with our results, other studies have reported that DNA methylation is known to change throughout aging and has been associated with age-related diseases including cancer, cardiovascular diseases, and atherosclerosis (Nebbioso et al., 2018; Salameh et al., 2020).

When interpreting our work, the strengths and limitations need to be considered. Firstly, we showed the effects of ART on the respiratory health and associated molecular mechanisms from a young age to elderly life to provide insights into the long-term respiratory health of an ART offspring. In addition, we adjusted for embryo transfer and litter size in our study; these factors themselves have been detected to increase the risk of diseases in an ART-born offspring later in life. Although every effort has been made, there are still limitations in our work. One limitation is that our study cannot draw conclusions on how the methylation patterns of these genes relate to changes in lung function throughout life which still requires further investigation at the cytological level. Moreover, this is an animal model study. Long-term studies in humans who were conceived by ART are necessary to determine the effects of ART on respiratory diseases later in life. Finally, the small size is another limitation in our study. Taken together, there is still a need for more works to show the effects of ART on lung diseases later in life and the associated molecular mechanisms in ART-born individuals.

## Data Availability Statement

The raw data supporting the conclusions of this article will be made available by the authors, without undue reservation.

## Ethics Statement

The animal study was reviewed and approved by the Zhejiang University Animal Care Committee.

## Author Contributions

FL and HL designed the study. FL, NW, and LW established the mouse models and performed the statistical analysis. XY and XL performed the gene expression analysis. QW, MH, and LL performed Western blot analysis and pyrosequencing. FJ revised the manuscript. All authors contributed to the article and approved the submitted version.

## Conflict of Interest

The authors declare that the research was conducted in the absence of any commercial or financial relationships that could be construed as a potential conflict of interest.

## Supplementary Material

Supplementary Figure 1 Expression level analysis of nSREBP associated genes in the lungs from the ICSI, IVF, and *in vivo* groups (*n* = 10/group). mRNA expression level analysis at 1.5 years of age by real-time quantitative PCR (RT-qPCR). **(A)** mRNA expression level of nSREBP targets genes of *Ldlr*, *Cyp51*, *Aacs*, *Fdps*, and *Hmgcr*. **(B)** mRNA expression level of *Mbtps1* and *Mbtps2*. The relative expression levels represent the amount of expression normalized to Gapdh expression. Data concerning the relative amount was calculated by the 2^–ΔΔ*Ct*^ method. Mean ± *SD* values are plotted. Between-group comparisons were made by one-way analysis of variance (ANOVA). ^∗∗^*P* < 0.01.

Supplementary Table 1 The genes sequences for real-time quantitative PCR.

Supplementary Table 2 The genes sequences for BSP and pyrosequencing.
