# Supplementary figures and images for: Long-Term Disturbed Expression and DNA Methylation of SCAP/SREBP Signaling in the Mouse Lung From Assisted Reproductive Technologies
Source: Front Genet. 2021 Jun 24;12:566168. doi: 10.3389/fgene.2021.566168 (PMC8266399; doi:10.3389/fgene.2021.566168)

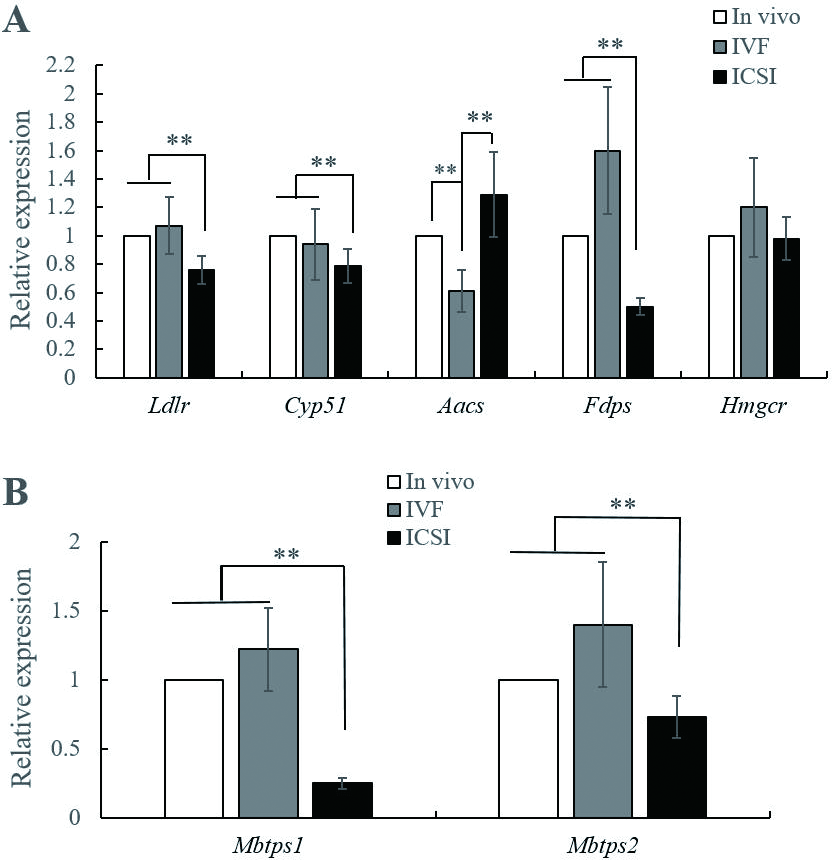

Supplement: Supplementary Figure 1 — Expression level analysis of nSREBP associated genes in the lungs from the ICSI, IVF, and in vivo groups (n = 10/group). mRNA expression level analysis at 1.5 years of age by real-time quantitative PCR (RT-qPCR). (A) mRNA expression level of nSREBP targets genes of Ldlr, Cyp51, Aacs, Fdps, and Hmgcr. (B) mRNA expression level of Mbtps1 and Mbtps2. The relative expression levels represent the amount of expression normalized to Gapdh expression. Data concerning the relative amount was calculated by the 2–ΔΔCt method. Mean ± SD values are plotted. Between-group comparisons were made by one-way analysis of variance (ANOVA). ∗∗P < 0.01. [file Image_1.tif]
