# Supplementary material for: Long-Term Disturbed Expression and DNA Methylation of SCAP/SREBP Signaling in the Mouse Lung From Assisted Reproductive Technologies
Source: Front Genet. 2021 Jun 24;12:566168. doi: 10.3389/fgene.2021.566168 (PMC8266399; doi:10.3389/fgene.2021.566168)
Supplement: Supplementary Table 1 — The genes sequences for real-time quantitative PCR. [file Data_Sheet_1.doc]

**Long-term disturbed expression and DNA methylation of SCAP/SREBP signaling in the mouse lung from assisted reproductive technologies**

Fang Le^1^, Quanmin Kang^1^, Ning Wang^1^, Qijing Wang^1^, Xinyun Yang^1^, Lejun Li^1^, Liya Wang^1^, Xiaozhen Liu^1^, Minhao Hu^1^, Fan Jin^1, 2^ and Hangying Lou^1^*

^1^ Center of Reproductive Medicine, Zhejiang University School of Medicine Women's Hospital, Hangzhou, China; ^2^ Key Laboratory of Reproductive Genetics, Ministry of Education, Hangzhou, China

**Supplemental table 1. The genes sequences for real-time quantitative PCR**

| **Genes** **and GenBank**  **Accession number** | **Primer sequence (5’-3’)** | | **Product**  **size (bp)** |
| --- | --- | --- | --- |
| *Scap* ([NM_001001144.2](http://www.ncbi.nlm.nih.gov/sites/entrez?cmd=search&db=nucleotide&dopt=GenBank&term=NM_001001144.2)) |  | | 173 |
| Forward | GCATCACAGCCCTTGTCTTC | |  |
| Reverse | CACTGTGTTGCTGCTGCTGTA | |  |
| *Srebf2* ([NM_033218.1](http://www.ncbi.nlm.nih.gov/sites/entrez?cmd=search&db=nucleotide&dopt=GenBank&term=NM_033218.1)) |  |  | 138 |
| Forward | GTCGATCAAGTCAGCAGCCAAG | |  |
| Reverse | TTGGCCTGAGGTTTCACCAAG | |  |
| *Ldlr* (NM_001252659) |  | | 116 |
| Forward | GATGGCTATACCTACCCCTCAA | |  |
| Reverse | TGCTCATGCCACATCGTC | |  |
| *Aacs* (NM_030210)  Forward  Reverse | GGGAGCCTGACAGCAAGAAG  CGGACAGACCAGTGGTATAAGTC | | 145 |
| *Cyp51* (NM_020010)  Forward  Reverse  *Fdps* (NM_134469) | AACGAAGACCTGAATGCAGAAG  GTGGGCTATGTTAAGGCCACT | | 138  130 |
| Forward  Reverse  *Hmgcr* (NM_013134)  Forward  Reverse  *Mbtps1* (NM_001167910)  Forward  Reverse  *Mbtps2* (NM_172307)  Forward  Reverse | ATGCCATCAACGACGCTCTG  CCGATCTCTGTCTGATAGGAACT  CACGCTCATAGTCGCTGGAT  TGACAGCCAAAAGGAAGGCT  TTGTGGCTTTCAACGGATACTT  AGGGTAGTCACTGGATGGGTT  TAATCGTGCCTTTTACAGTTGGG  CATGGCAATCACGCCAAACA | | 81  125  91 |
| *Gapdh* (NM_008084.2) |  | | 150 |
| Forward | TGTGTCCGTCGTGGATCTGA | |  |
| Reverse | TTGCTGTTGAAGTCGCAGGAG | |  |

**Supplemental table 2**. The genes sequences for BSP and pyrosequencing

| **Genes** | **Primer sequence (5’-3’)** | **PCR (bp)** | | **Pryosequencing primers (5’-3’)** | **CpG numbers** | |
| --- | --- | --- | --- | --- | --- | --- |
|
| *Scap*  Forward | ATTTAGGAGAATTGGGAAAGGTT | 184 | | GAAGAATTTGGAGATAGTTT | 6 | |
| Reverse-biotin | AAATAAACTCTAAAAACAACTACACC | | | | | |
|  |  |  |  | | |  |
| *Srebf1* | |  |  | | | 4 |
| Forward | TAAAGGTTTTTAAGGGAAAGTTTTG | 190 | GGGTTTAGTTGGAAGTG | | |  |
| Reverse-biotin | ACTATCTACCCCCTACCTTCCTATC | | | | | |
|  |  |  |  | | |  |
| *Srebf1* | |  |  | | | 5 |
| Forward-biotin | TAAAGGTTTTTAAGGGAAAGTTTTG | 190 | AAACAACGAATAAACATACT | | |  |
| Reverse  *Srebf2*  Forward  Reverse-biotin  *Srebf2* Forward-biotin  Reverse | ACTATCTACCCCCTACCTTCCTATC  5  TTTTTAAAGTTTTGTAGTTTTAAGTGTAAA 224 TATATTGTAGATTTTTGTTT  AAACTAATTCAAACTCCTTTCCAAAC  3  TTTTTAAAGTTTTGTAGTTTTAAGTGTAAA 224 ACAATATACCATTAACTATC  AAACTAATTCAAACTCCTTTCCAAAC | | | | | |
